# Supplementary material for: Knowledge, attitude, and practice towards Hepatitis B and vaccination status of pre-clinical medical students at Sylhet Women’s Medical College, Bangladesh
Source: PLoS One. 2024 Nov 25;19(11):e0310443. doi: 10.1371/journal.pone.0310443 (PMC11588231; doi:10.1371/journal.pone.0310443)
Supplement: Attachment — Submitted filename: Response to comments.docx [file pone.0310443.s001.docx]

**RESPONSE TO ACADEMIC EDITOR:**

1. Please ensure that your manuscript meets PLOS ONE's style requirements, including those for file naming.

- Thank you for this comment
- We have edited as required in the manuscript.

2. Please provide additional details regarding participant consent. In the ethics statement in the Methods and online submission information, please ensure that you have specified (1) whether consent was informed and (2) what type you obtained (for instance, written or verbal, and if verbal, how it was documented and witnessed).

- Thank you for this comment
- Informed written consent was taken from each participant. We have edited as required in the manuscript.

3. Please note that your Data Availability Statement is currently missing the DOI/accession number of each dataset or a direct link to access each database. If your manuscript is accepted for publication, you will be asked to provide these details on a very short timeline. We therefore suggest that you provide this information now, though we will not hold up the peer review process if you are unable.

- Thank you for this suggestion
- We have added DOI link of figshare data in the manuscript as required.

4. Please upload a copy of Supporting Information which you refer to in your text on page 26.

- Thank you for this suggestion
- We have uploaded the copy of supporting information as referred in the text (figshare doi link: https://doi.org/10.6084/m9.figshare.25845664.v1).

**RESPONSE TO REVIEWER-1:**

1. For questions- "vaccination against hepatitis B- yes or no" or "dose of HBV vaccine received- 1,2,3, 3 +booster?", was there an option indicating "i don't know" or an option to skip? This important as 43% of participants were "not aware of their hepatitis B vaccine status" so it is likely that 43% of the participants answered the 2 questions inaccurately. Recall bias is a big limitation of the study, which the authors do mention in the limitation section. They also mention that serological methods to confirm vaccination status would have been ideal to overcome this. However, I would recommend describing it more, including possible steps the authors took to minimize this bias in the study.

- Thank you for these comments. Actually, the question “reason for not being vaccinated against Hepatitis B” was applicable for those who responded ‘NO’ to vaccination. And, this was the most commonly reported cause of non-vaccination among the participants.
- We have tried to explain more on methods that can be used to reduce recall bias in this type of study. We have edited this in the DISCUSSION section of the manuscript.

2. Was pre-testing/pilot testing performed? Some questions can possibly be interpreted in ways the authors did not indent/ not be clear (eg: Lack of accessibility for Hepatitis B vaccine, not aware of the Hepatitis B vaccine status). Also were any tests of validity of the questionnaire performed prior to the survey?

- Thank you for this comment. These questionnaires were adapted from Shrestha DB et. al, 2020 with permission. This study was conducted in Nepal with pre-testing done in 5% of the study sample. So, considering the similar population characteristics in South Asian region, we did not perform pre-testing in our sample.
- However, we did assess internal consistency of our questionnaires that showed a good internal consistency, with a Cronbach's alpha of 0.71.

3. Authors did a good work with literature review in discussion but also describe the clinical/real-world implications of the results in the discussion.

- Thank you for your compliment and comment. We have edited in the DISCUSSION section of the manuscript, where we have tried to describe clinical implications of our results.

4. Describe briefly the informed consent process- were participants told of what data collected and stored, who the investigators are, the purpose of study, etc. What personal information was collected?

- Thank you for this comment. We have explained this information in detail in methods section in the manuscript.

5. Were any steps taken to prevent multiple entry of data from a single participant? How was it ensured that each entry of the survey was a unique participant?

- Thank you for this concern. We had sent the google forms to each participant in their social media platform (facebook, whatsapp, email, etc). Each form was designed to accept only one unique response, preventing resubmission once finished. So, there was no possibility of multiple entry of data from a single participant.

6. Briefly mention what is the guideline for HBV vaccination in Bangladesh- childhood vaccination, healthcare workers? If it was part of childhood immunization program for more than 20 years, it is unlikely that 66% were not vaccinated (unless immunization coverage/ compliance is poor in the country)

- Thank you for this comment. We have added this information in DISCUSSION section of the manuscript.

7. I would add lack of generalizability/ ext validity in limitation- as study only included 1st and 2nd year medical students, and also 1 medical college in the country.

- Thank you for this comment. We totally agree with you that there is lack of generalizability/external validity in our study due to inclusion of pre-clinical medical students of one medical college only. We have added this in our manuscript.

8. Minor points

- Line 280- sample size is not small as the entire population (1st and 2nd yr students at the medical college) was sampled.

- line 82- this is not "non-probability convenient sampling method" as the whole population (all 1st and 2nd year students of the medical college) was used as sample.

- Table 1. 5th row, 3rd column- should be 21 (20-21)

- Table 5- present as n(%)

- Thank you for these valuable suggestions. We have edited accordingly in the manuscript.

**RESPONSE TO REVIEWER-2:**

1. Background needs to better demonstrate the novelty of this resource, and add more information on how is hepatitis B vaccine given in their nation (for free or charge).

- Thank you for this comment.
- Studies of this nature are commonly conducted worldwide. However, given that Bangladesh is situated in an intermediate endemic zone with lower Hepatitis B vaccination rates compared to other Southeast Asian nations, this research holds significant clinical and administrative importance. While the topic may lack novelty, it remains practically important from a public health perspective, as medical students represent a vulnerable population with respect to Hepatitis B infection.
- We have added the information related to Hepatitis B vaccination in Bangladesh in DISCUSSION section in the manuscript.

2. Methods are unclear about recruitment and survey procedures.

- Thank you for this comment
- We adopted whole sampling technique, i.e. included all the pre-clinical medical students of a medical college. A questionnaire was sent to individual participant in the form of Google forms via different social media (facebook, whatsapp, email), and their responses were recorded in Excel form. The first page of the Google form consists of background and objectives of the study, authors’information and consent options. Those who consented were forwarded to next pages which consist of actual questionnaires. All these responses were accurately recorded subsequently in Excel form which was later used for data analysis and interpretation. We have tried to explain this in the manuscript in more elaborative way. Please kindly consider it.

3. The sampling technique and sample size are not clear yet. Explain how the study size was arrived at

- Thank you for this comment
- We used whole sampling technique which included all the pre-clinical medical students (MBBS 1st and 2^nd^ academic years), i.e. Sample size = Total population

4. Define all outcomes and give details of methods of assessment. In addition, the authors need to provide the cut-off level of good knowledge, attitude and practice, and references. For assessing KAP of the previous studies, knowledge, and practice variables are often classified as correct and incorrect, and attitude is assessed by using a five-point Likert scale and classified as positive and negative, the authors need to consider this. For KAP studies, the variable definition is not accurate, the results are less valid and reliable.

- Thank you for this comment and suggestions.
- These questionnaires were adapted from Shrestha DB et. al, 2020 with permission.^1^ Similar pattern of scoring was used in our study as well. We have edited an explanation about KAP scoring and different variables in the manuscript itself.

5. The questionnaire contained 5 for knowledge as described in the methods, the authors can explain how to calculate the knowledge sum of 54.01±7.57 (table 3)

- Thank you for this suggestion.
- We have explained this information in the manuscript. Actually, total score was calculated by adding individual score (based on Likert scale) on each item in knowledge, attitude and practice section.

6. The authors need to present the association between baseline characteristics and knowledge, attitude, and practice separately, not combining them (Table 5)

- Thank you for this suggestion
- Exploring association between baseline characteristics and knowledge, attitude and practice separately is a very good approach but it does not fall under our research objectives. Instead, we have studied the association between overall score (categorical) and baseline characteristics. It is because this association has been shown by a reference study by Shrestha DB et al,^1^ where association has been demonstrated between total KAP score category and baseline characteristics.

**REFERENCE**

1. Shrestha DB, Khadka M, Khadka M, Subedi P, Pokharel S, Thapa BB. Hepatitis B vaccination status and knowledge, attitude, and practice regarding Hepatitis B among preclinical medical students of a medical college in Nepal. PLoS One [Internet]. 2020 Nov 1 [cited 2024 Mar 12];15(11):e0242658. Available from: https://journals.plos.org/plosone/article?id=10.1371/journal.pone.0242658
