# Supplementary material for: Knowledge, attitude, and practice towards Hepatitis B and vaccination status of pre-clinical medical students at Sylhet Women’s Medical College, Bangladesh
Source: PLoS One. 2024 Nov 25;19(11):e0310443. doi: 10.1371/journal.pone.0310443 (PMC11588231; doi:10.1371/journal.pone.0310443)
Supplement: Attachment — Submitted filename: Comments.docx [file pone.0310443.s002.docx]

This study is well written with appropriate use of the statistical methods for the analysis of data, the results are briefed and reasoned appropriately. There are some minor and some major mistakes or missing that need to be corrected with some grammatical editing with rephrasing of the sentences. With these corrections, this article will be acceptable for the publications.

**Minor:** I recommend using the “pre-clinical medical student” throughout the manuscript to resolve the confusion associated with use of “pre-clinical medical student”, “medical student” or “health science students”.

Need grammatical corrections throughout the manuscript to make it easily comprehendible for the reader. You may take help of English language expert.

**Abstract:**

In materials and methods: “An online, single-center, descriptive cross-sectional study”: Use web-based rather than on “online”

In result: instead of only vaccine, mention the vaccine being Hepatitis B vaccine

**Manuscript**

**Method:**

- Reason for the choosing pre-clinical medical students not clear and also the risk of exposure to hepatitis B among participants not mentioned, like whether they have exposure to clinical practice or not, if yes, how’s the risk of the hepatitis B as per hospital record on needle injury among targeted participant group
- Reason for the selection of whole sampling method need to be explained
- Need to acknowledge about the participants not giving consent, whether they were included or excluded

**Discussion:**

- “Moreover, pre-clinical medical students are less experienced and frequently sustain needle-stick injuries while performing different procedures with infected patients.” Need reference for this line and whether it applies to participants of this study or not.
- Findings of study were compared to findings from other studies without mentioning whether they have similar cohort of participants or different.
