# Supplementary material for: Knowledge, attitude, and practice towards Hepatitis B and vaccination status of pre-clinical medical students at Sylhet Women’s Medical College, Bangladesh
Source: PLoS One. 2024 Nov 25;19(11):e0310443. doi: 10.1371/journal.pone.0310443 (PMC11588231; doi:10.1371/journal.pone.0310443)
Supplement: Attachment — Submitted filename: RESPONSE TO REVIEWERS.docx [file pone.0310443.s003.docx]

**RESPONSE TO REVIEWERS’COMMENTS:**

1. I recommend using the “pre-clinical medical student” throughout the manuscript to resolve the confusion associated with use of “pre-clinical medical student”, “medical student” or “health science students”.

- Thank you for the suggestion. We have edited in the manuscript as appropriate.

2. Need grammatical corrections throughout the manuscript to make it easily comprehendible for the reader. You may take help of English language expert.

- Thank you for the valuable suggestion. We have tried our best to edit the manuscript accordingly.

3. In materials and methods: “An online, single-center, descriptive cross-sectional study”: Use web-based rather than on “online”

- Thank you for the suggestion. We have edited accordingly.

4. In result: instead of only vaccine, mention the vaccine being Hepatitis B vaccine

- Thank you for the suggestion. We have edited accordingly.

5. Reason for the choosing pre-clinical medical students not clear and also the risk of exposure to hepatitis B among participants not mentioned, like whether they have exposure to clinical practice or not, if yes, how’s the risk of the hepatitis B as per hospital record on needle injury among targeted participant group.

- Thank you for this comment. Our study sample consists of pre-clinical medical students who are moving into their clinical phase in near future. The pre-clinical medical students have little experience and during their clinical posting, they are apparently exposed to infected patients. Moreover, they have to get involved in different types of invasive procedures putting them at a high risk of needle-stick injuries. So, this group of students is at high risk of acquiring Hepatitis B infection. We have described these things in detail with relevant references in our INTRODUCTION and DISCUSSION section. And, we have made a few edits in the manuscript as appropriate. Please kindly consider it.

6. Reason for the selection of whole sampling method need to be explained

- Thank you for this comment. In our study, we have included all the medical students in their pre-clinical academic years (i.e. whole sampling method). This means, we have included the entire study population (i.e. pre-clinical medical students of a medical college) in this research. It helps to decrease selection bias, sampling error, and increase the accuracy of our findings. We have added this information in DISCUSSION section last paragraph.

7. Need to acknowledge about the participants not giving consent, whether they were included or excluded

- Thank you for this valuable suggestion. We have edited this information in METHODS section of the manuscript.

8. “Moreover, pre-clinical medical students are less experienced and frequently sustain needle-stick injuries while performing different procedures with infected patients.” Need reference for this line and whether it applies to participants of this study or not.

- Thank you for this comment. We have cited references 4, 5, 12, 13 for all these information in paragraph 1 of DISCUSSION. Since this is an integrated information, we thought referencing as a whole would suit better. Please consider it.

9. Findings of study were compared to findings from other studies without mentioning whether they have similar cohort of participants or different.

- Thank you for this comment. We totally agree with your opinion regarding our study findings being compared with other studies without mentioning the cohort characteristics. Most of the time, we have tried to compare and contrast our results with the studies from South East Asia region where there are approximately similar socio-economic and educational characteristics. However, there were differences in sample size, sampling method, gender distribution, and other baseline characteristics. All those studies were observational studies (cross-sectional type), so we think that this difference should not matter much with our reporting in DISCUSSION section. Basically, we tried to compare our findings of vaccination rate and possible barriers to vaccination with other countries of the world (both developed and developing). Please kindly consider it.
